# Application of Schroth exercise therapy in adolescent idiopathic scoliosis: a bibliometric analysis

**DOI:** 10.3389/fspor.2026.1872364

**Published:** 2026-09-14

**Authors:** Chenlu Rao, Jinming Wang, Meiyuan Hu, Yan Yang, Wanfen Song, Wenjuan Zhou, Yuanyuan Mi

**Affiliations:** 1 Medical Department, Yangtze University, Jingzhou, Hubei, China; 2 Operating Room Area, Union Hospital, Tongji Medical College, Huazhong University of Science and Technology, Wuhan, Hubei, China; 3 Department of Orthopedics, Union Hospital, Tongji Medical College, Huazhong University of Science and Technology, Wuhan, Hubei, China; 4 Department of Critical Care Medicine, the Sixth Hospital of Wuhan, Affiliated Hospital of Jianghan University, Wuhan, China; 5 Evidence-based Nursing Research Center, the Sixth Hospital of Wuhan, Affiliated Hospital of Jianghan University, Wuhan, China

**Keywords:** adolescent idiopathic scoliosis, author co-authorship network, bibliometrics, keyword clustering, physiotherapeutic scoliosis-specific exercises, Schroth exercise

## Abstract

**Background:**

Adolescent idiopathic scoliosis (AIS) is a common three-dimensional spinal deformity. Schroth exercise, a form of physiotherapeutic scoliosis-specific exercise (PSSE), has increasingly been studied in relation to Cobb angle, angle of trunk rotation (ATR), and health-related quality of life. However, the publication landscape, thematic evolution, influential contributors, and collaboration networks of this field have not been comprehensively characterized.

**Objective:**

To map publication output, collaboration networks, influential documents, institutions, journals, authors, and thematic evolution in research on Schroth exercise therapy for AIS.

**Methods:**

English-language literature published up to April 1, 2026, was retrieved from the Web of Science Core Collection (WoSCC), PubMed, and Scopus. After screening, 172 publications were included. The bibliometrix package (version 5.0) and CiteSpace 6.4.R1 were used to analyze country collaboration and publication output, global citation counts, institutional and journal publication output, journal dual-map citation pathways, keyword clusters, citation bursts and timelines, author co-citation, and author co-authorship networks.

**Results:**

China had the highest publication output, while the United States and Germany were tied for second. China had the highest betweenness centrality, followed by Germany and Canada. The University of Alberta, Guangzhou Sport University, and The University of Hong Kong were the leading conventional research institutions. Negrini S, Kuru T, and Schreiber S were the most frequently co-cited authors, whereas Hedden, Douglas M., Schreiber, Sanja, and Parent, Eric C. were among the leading publishing authors. Keyword analyses indicated an evolution from broad conservative management toward Schroth-specific approaches and outcome-oriented evaluation, with recent attention to ATR, PSSE, and home-based exercise.

**Conclusion:**

Schroth exercise therapy has become an important focus within the nonsurgical management literature for AIS. This bibliometric analysis characterizes publication trajectories, academic networks, influential contributors, and evolving research themes. Conclusions regarding clinical efficacy should be derived from the underlying clinical trials and evidence syntheses rather than from the bibliometric design itself.

## 1 Introduction

Adolescent idiopathic scoliosis (AIS) is the most common spinal deformity arising during adolescence, typically between 10 and 18 years of age. It is characterized by a coronal Cobb angle of at least 10° together with vertebral rotation, constituting a complex three-dimensional deformity (1). Its etiology remains unclear and is considered multifactorial, involving genetic, hormonal, neural, skeletal-growth, and biomechanical influences (2). The estimated prevalence among children and adolescents is approximately 1%–4%, with a greater risk of clinically significant progression among females (3, 4). AIS may lead to postural asymmetry, pain, functional limitations, psychosocial stress, and reduced quality of life; severe or untreated major curves may also be associated with chest-wall deformity and impaired pulmonary function (1, 5, 6). These potential consequences highlight the importance of early detection and appropriate intervention to reduce progression and long-term adverse outcomes.

The Schroth method is a scoliosis-specific physiotherapeutic approach that has received increasing attention in conservative AIS management. It emphasizes three-dimensional postural correction, rotational breathing, active self-correction, and functional stabilization. Through curve-pattern-specific muscle activation and postural training, Schroth exercise aims to improve spinal alignment, trunk control, and functional stability (7, 8). It belongs to the broader category of physiotherapeutic scoliosis-specific exercises (PSSE), which integrate three-dimensional self-correction, training in activities of daily living, and respiratory components to improve postural control and muscular coordination (9). Compared with other PSSE approaches, such as the Scientific Exercise Approach to Scoliosis (SEAS), the Schroth method uses a distinct curve-classification system and rotational-breathing techniques to support individualized treatment planning (10).

Although international guidelines, including those of the Society on Scoliosis Orthopaedic and Rehabilitation Treatment (SOSORT), recommend physiotherapy as a component of conservative AIS management, evidence for specific interventions such as Schroth exercise remains heterogeneous, and high-quality studies are still needed to guide clinical practice (11, 12). Recent clinical studies and evidence syntheses have reported potential benefits of Schroth exercise. For example, a retrospective study reported reductions in Cobb angle and improvements in pain and functional scores after a Schroth-based intervention and also evaluated clinical and economic outcomes (13). A systematic review and meta-analysis reported that Schroth three-dimensional exercise may reduce Cobb angle and angle of trunk rotation (ATR), improve quality of life, and increase lumbar extensor strength compared with conventional physical therapy (14).

In addition, a network meta-analysis comparing several exercise therapies reported favorable rankings for Schroth therapy in outcomes related to spinal deformity and quality of life, thereby providing a systematic perspective on the relative performance of different exercise therapies (15).

Although broader bibliometric studies of AIS treatment have been published (16), clinical systematic reviews and meta-analyses have mainly synthesized intervention effects on outcomes such as Cobb angle, ATR, and health-related quality of life. They do not provide a dedicated landscape analysis of publication trajectories, global collaboration, influential documents, leading institutions and journals, thematic clusters, or author networks specific to Schroth exercise therapy. A bibliometric approach is therefore needed to characterize the knowledge structure and temporal development of this field.

Bibliometrics is a systematic quantitative approach for characterizing the development, knowledge structure, and temporal evolution of a research field. This study applies bibliometric analysis to the literature on Schroth exercise therapy for AIS using complementary visualizations. The literature screening flowchart summarizes retrieval, eligibility assessment, and inclusion; country collaboration and publication-output analyses describe national contributions and international cooperation; global citation analysis, institution and journal publication-output analyses, and a journal dual-map overlay characterize influential literature, productive organizations and publication venues, and disciplinary citation pathways; keyword clustering, burst, and timeline analyses identify thematic structures and their temporal evolution; and author co-citation and co-authorship networks distinguish intellectual influence from direct research collaboration.

Through these maps and tables, this study identifies influential publications, leading research institutions and journals, influential co-cited authors, active publishing authors, major research hotspots, temporal trends, and evidence gaps. The findings provide a structured bibliometric overview that may inform future multicenter clinical studies, international collaboration, and rehabilitation research.

## 2 Methods

### 2.1 Literature search

This study searched three databases: the Web of Science Core Collection (WoSCC), PubMed, and Scopus. The search covered each database from inception to April 1, 2026. Only English-language publications were considered, and no publication-year restriction was applied. The study design, data extraction, and visual analyses were conducted in accordance with the minimum reporting requirements of the Bibliometric Review Reporting Guidelines in Biomedicine (BIBLIO) (17).

The search strategy comprised two concept blocks. The first addressed the study population, including adolescent idiopathic scoliosis, idiopathic adolescent scoliosis, AIS, and teenage scoliosis. The second addressed Schroth-based interventions, including the Schroth method, Schroth exercise, Schroth therapy, and Schroth physiotherapy. The two blocks were combined with the Boolean operator AND. PubMed used a combination of controlled vocabulary and keywords, WoSCC used Topic Search, and Scopus used the TITLE-ABS-KEY field. The complete database-specific search strings are presented in Figure 1.

**Figure 1:**
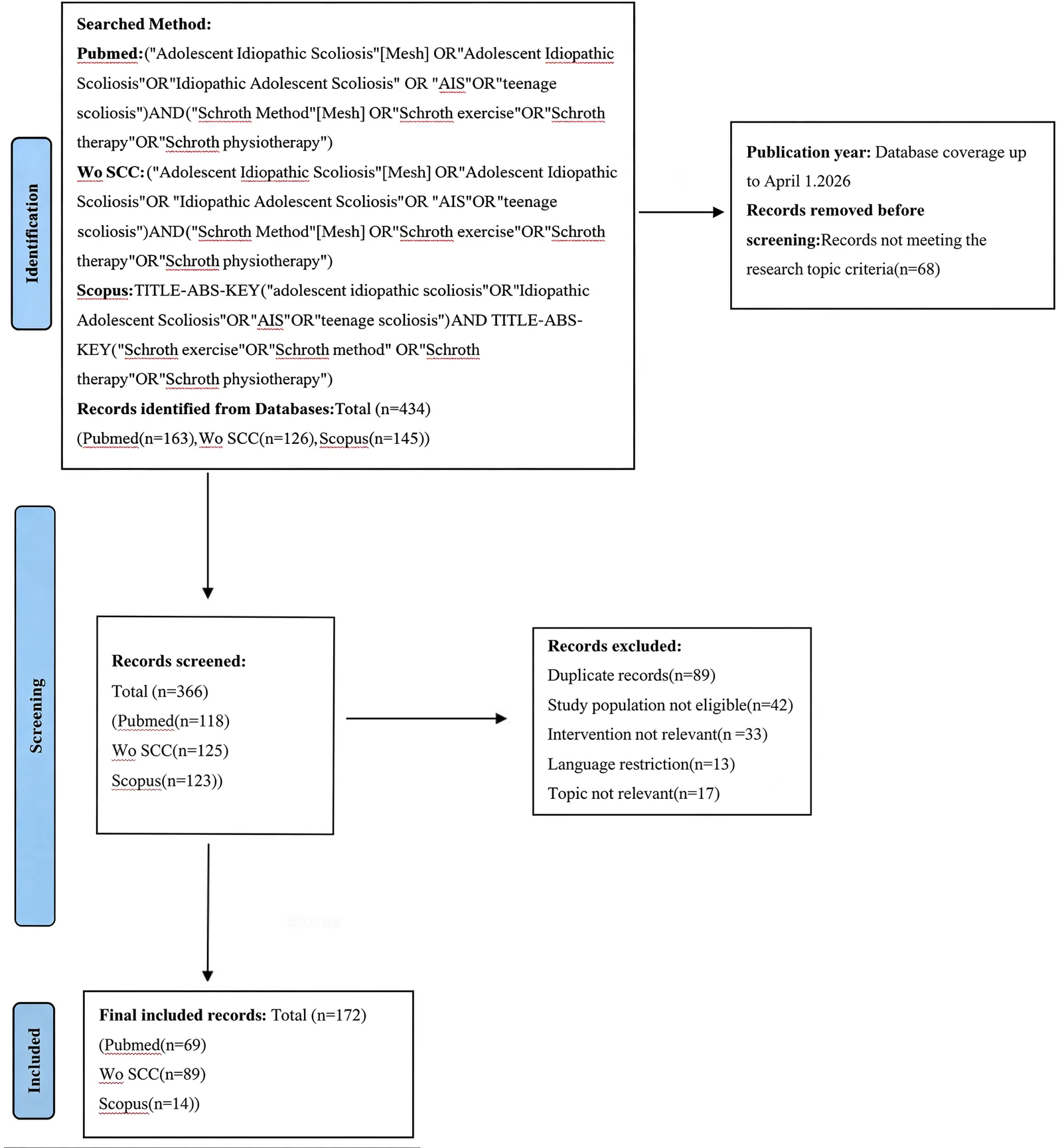
Flowchart of literature retrieval, screening, and inclusion.

### 2.2 Literature screening

The search identified 434 records: 163 from PubMed, 126 from WoSCC, and 145 from Scopus. Before formal screening, 68 records that did not meet the research-topic criteria were removed, leaving 366 records for screening. A further 194 records were excluded because they were duplicates (*n* = 89), involved an ineligible population (*n* = 42), evaluated an irrelevant intervention (*n* = 33), were non-English publications (*n* = 13), or were otherwise unrelated to the topic (*n* = 17). The final dataset comprised 172 publications: 69 from PubMed, 89 from WoSCC, and 14 from Scopus (Figure 1).

### 2.3 Inclusion and exclusion criteria

Inclusion criteria:

1. The study population consisted of adolescents with idiopathic scoliosis (AIS), aged 10–18 years;
2. The intervention involved Schroth exercise therapy or a Schroth-based physiotherapeutic scoliosis-specific exercise program;
3. The study types included randomized controlled trials (RCTs), cohort studies, systematic reviews, meta-analyses, and bibliometric studies.

Exclusion criteria:

1. Literature unrelated to the topic (e.g., basic mechanism studies or animal experiments);
2. Case reports, conference abstracts, or articles without full texts;
3. Studies with incomplete data or lacking extractable key outcomes;
4. Non-English publications.

The screening and inclusion process was independently conducted by two researchers. Disagreements were first resolved through discussion; if consensus could not be reached, a third researcher was consulted to make a final decision.

### 2.4 Data extraction

Bibliographic information extracted from the included publications comprised title, authors, publication year, journal, total citation count, author affiliations, countries, abstract, keywords, cited references, and DOI. Clinical study characteristics and outcome measures, including Cobb angle, ATR, and health-related quality of life, were consulted only to verify the clinical context of bibliometric themes; no *de novo* meta-analysis was performed. Missing or incomplete bibliographic fields were checked against journal websites and cross-references where possible.

### 2.5 Data processing and bibliometric analysis

Records exported from the three databases were merged, and duplicate entries were removed using available bibliographic metadata before analysis. Author, institution, country, journal, and keyword fields were checked for obvious variants. Institution names were standardized where possible; Egyptian Knowledge Bank (EKB) was retained as an institution-related metadata entry because it appeared in the source data. The bibliometrix package (version 5.0) was used for country collaboration and publication-output analyses, global citation analysis, institution and journal publication-output analyses, and the journal dual-map overlay. Figure 4 reports the global citation counts returned by bibliometrix for the merged dataset as of April 1, 2026. CiteSpace 6.4.R1 was used for keyword co-occurrence and clustering, citation-burst detection, keyword timeline analysis, author co-citation, and author co-authorship analysis.

CiteSpace analyses covered 1991–2026 with one-year time slices and g-index selection (k = 25). Minimum spanning tree pruning was applied to the keyword clustering and timeline networks, whereas no pruning was applied to the author co-citation and co-authorship networks. Depending on the analysis, node size represents occurrence frequency, co-citation frequency, or publication output; links represent co-occurrence, co-citation, or co-authorship relationships; and betweenness centrality indicates the extent to which a node bridges different parts of a network.

## 3 Results

### 3.1 Country collaboration and publication output

Figure 2 shows the international collaboration network in research on Schroth exercise therapy for AIS. Darker country shading indicates greater publication output, while links represent international co-authorship relations. The United States showed relatively dense collaborative links, particularly with China and several European countries, and was therefore interpreted as an active contributor in terms of publication output and collaborative activity rather than as the country with the highest network centrality.

**Figure 2:**
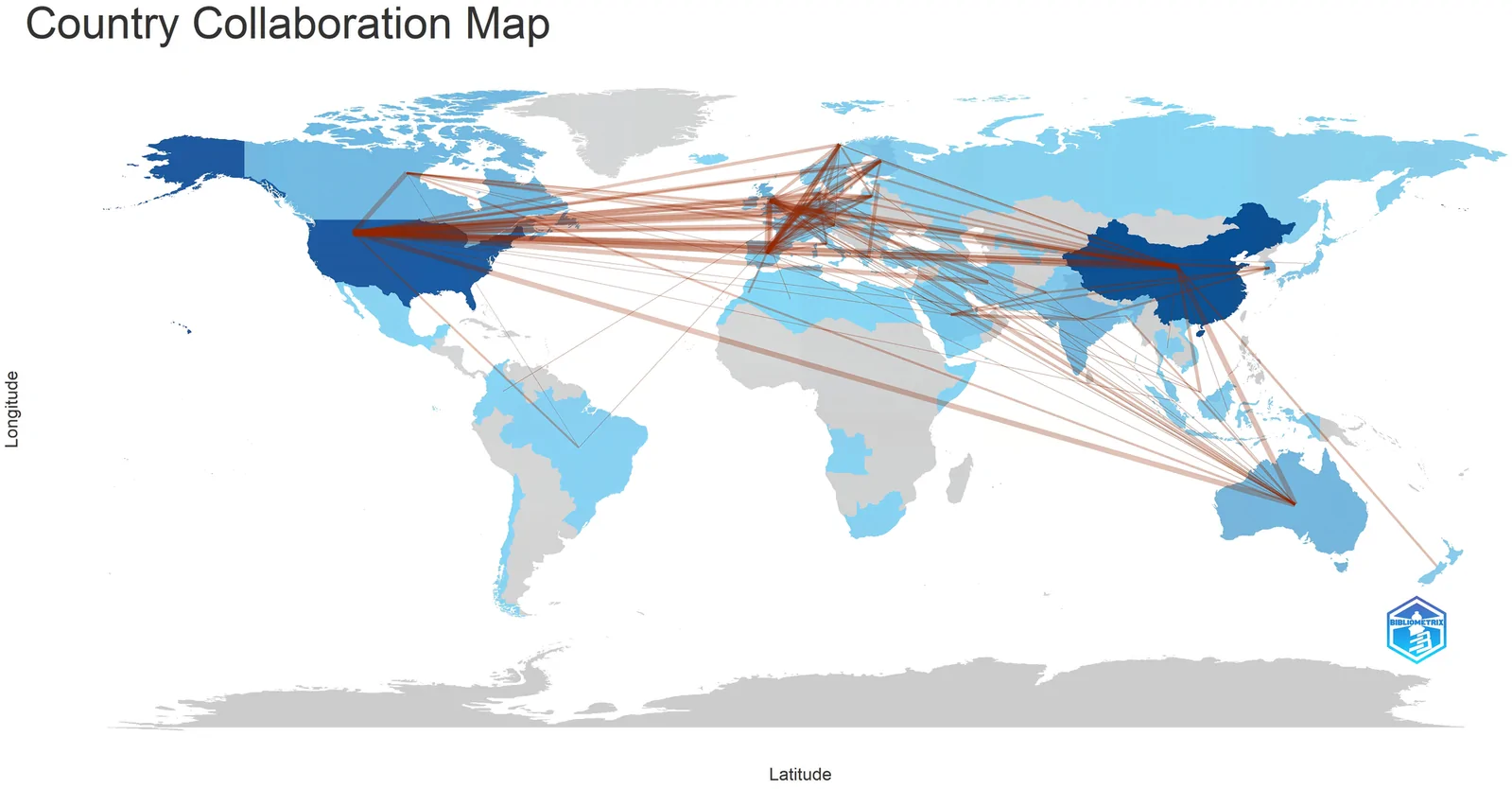
Country collaboration map.

As shown in Figure 3, China ranked first in publication output with 46 publications and had the highest betweenness centrality (0.16). The United States and Germany were tied for second with 18 publications each; however, Germany had a higher centrality value (0.11) than the United States (0.07). Turkey contributed 16 publications, while Canada contributed 11 publications and had a centrality value of 0.11. Poland and Iran contributed 6 publications each, and Australia, Greece, and Spain contributed 5 publications each. Betweenness centrality was interpreted as an indicator of a country's bridging role in the collaboration network (18). Publication output, collaboration intensity, and network centrality therefore represent distinct dimensions of country-level contribution.

**Figure 3:**
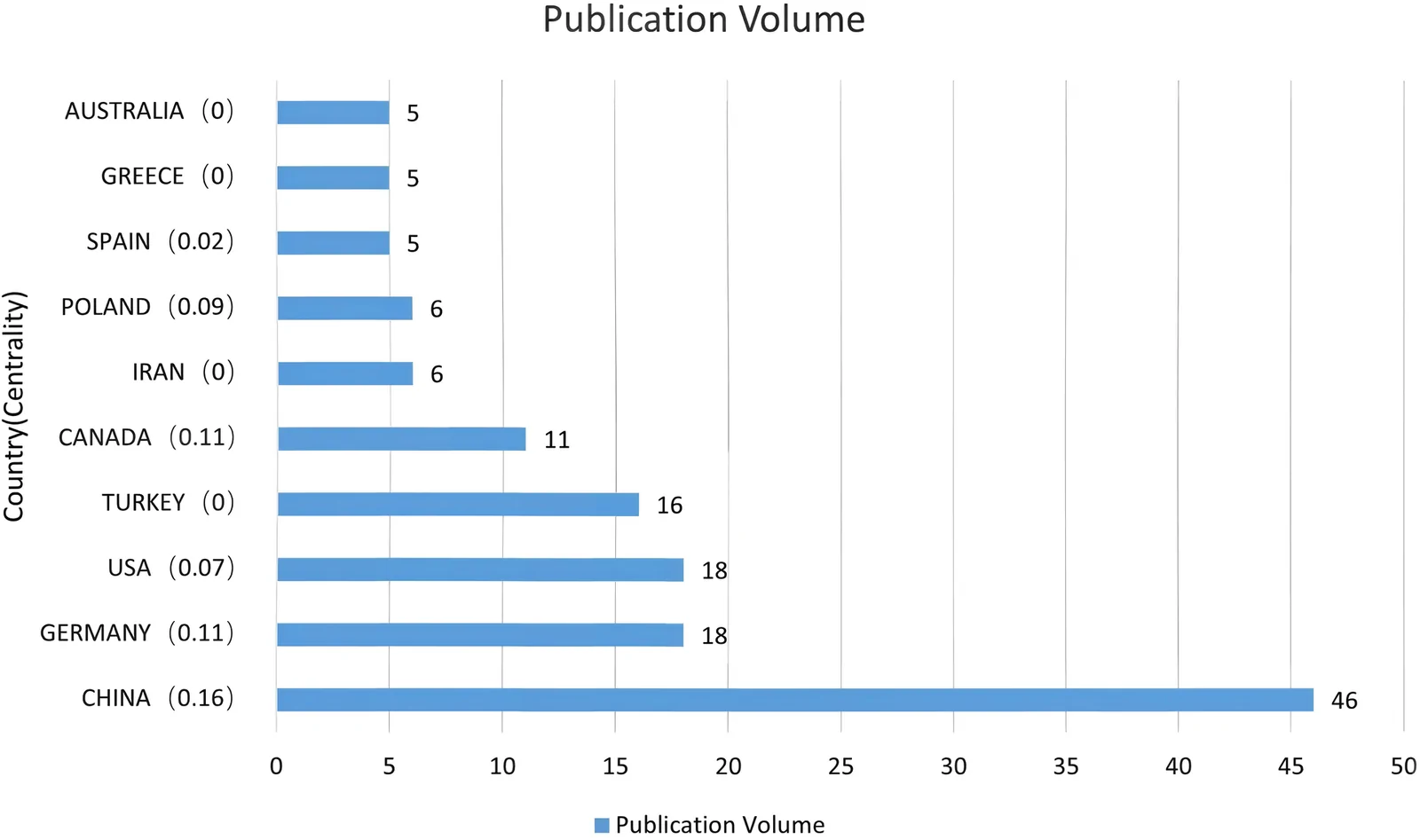
Publication output and centrality by country.

### 3.2 Analysis of highly cited documents, leading institutions, and leading journals

As shown in Figure 4, Kuru et al. (2016, Clinical Rehabilitation) (19) ranked first by global citation count, with 172 citations, followed by Schreiber et al. (2016, PLOS ONE) (20), with 128 citations. Other highly cited documents included Kocaman et al. (2021, PLOS ONE) (21), with 77 citations; Fan et al. (2020, BMC Musculoskeletal Disorders) (22), with 61 citations; and Zhou et al. (2021, Complementary Therapies in Medicine) (23) and Gao et al. (2021, Chinese Medical Journal) (24), with 33 citations each. The remaining documents in the top-10 set were Gámiz-Bermúdez et al. (2022, Clinical Rehabilitation) (25), Ma et al. (2023, Physiotherapy) (11), Romano et al. (2024, Cochrane Database of Systematic Reviews) (26), and Weiss (1992, Physiotherapy) (27). Within the top-10 set shown in Figure 4, the two leading documents accounted for 50.1% of the combined global citations, indicating that citation impact within this subset was concentrated in a small number of influential publications.

**Figure 4:**
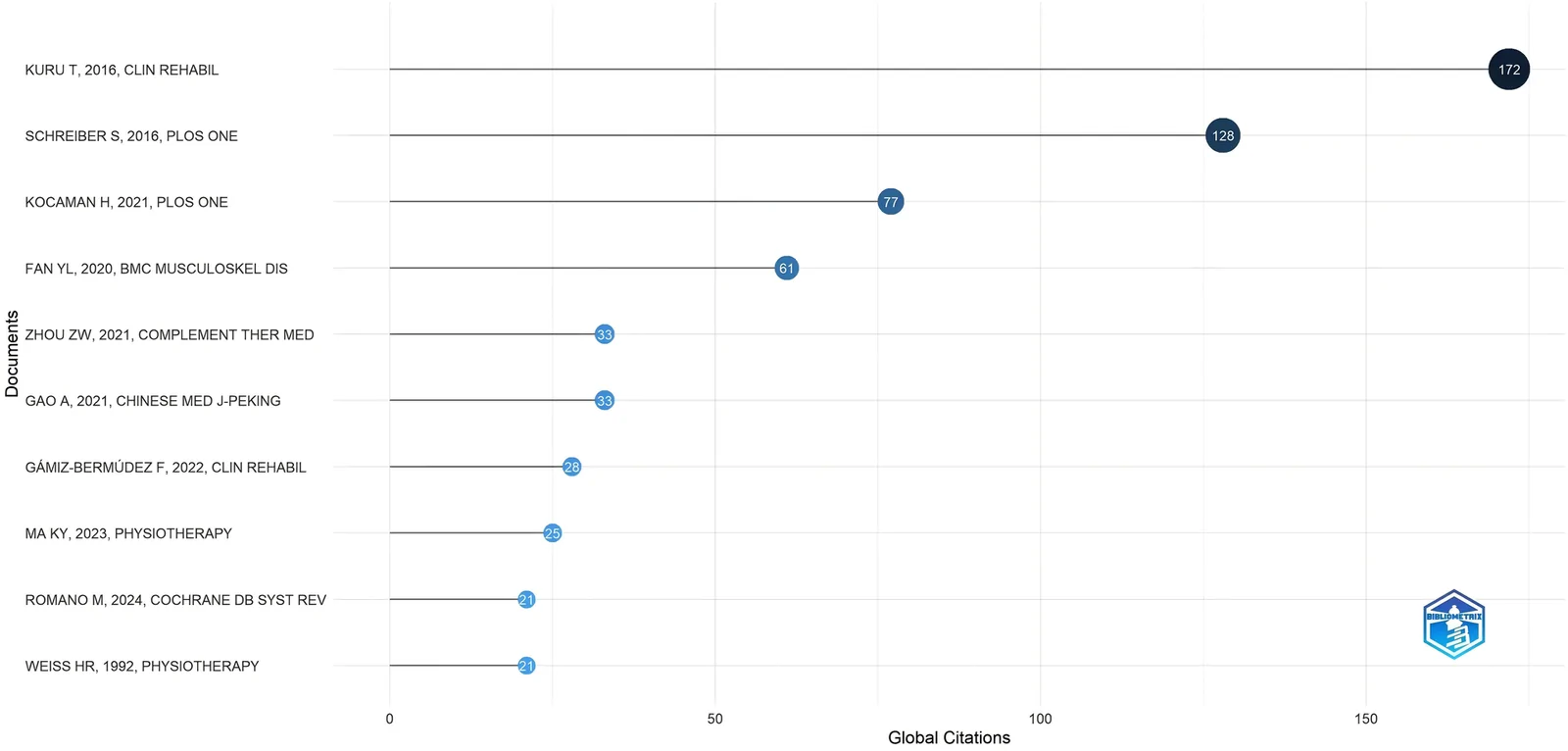
Top 10 documents by global citation count in research on Schroth exercise therapy for AIS.

Figure 5 presents the top 10 institutions ranked by publication output. The University of Alberta ranked first with 26 publications, followed by Guangzhou Sport University with 16 publications and The University of Hong Kong with 9 publications. Four institution-related entries had 8 records: Egyptian Knowledge Bank (EKB), International Hellenic University, Stellenbosch University, and the University of Tehran. EKB was retained because it appeared as an institution-related entry in the source metadata; however, because it is a knowledge platform rather than a conventional university, hospital, or research institute, it was not interpreted as an independent research institution when identifying the principal institutional contributors. Kirsehir Ahi Evran University, Kunming Municipal Hospital of Traditional Chinese Medicine, and Yibin Traditional Chinese Medicine Hospital each contributed 7 publications. Overall, universities and medical institutions from multiple countries and regions contributed to this field.

**Figure 5:**
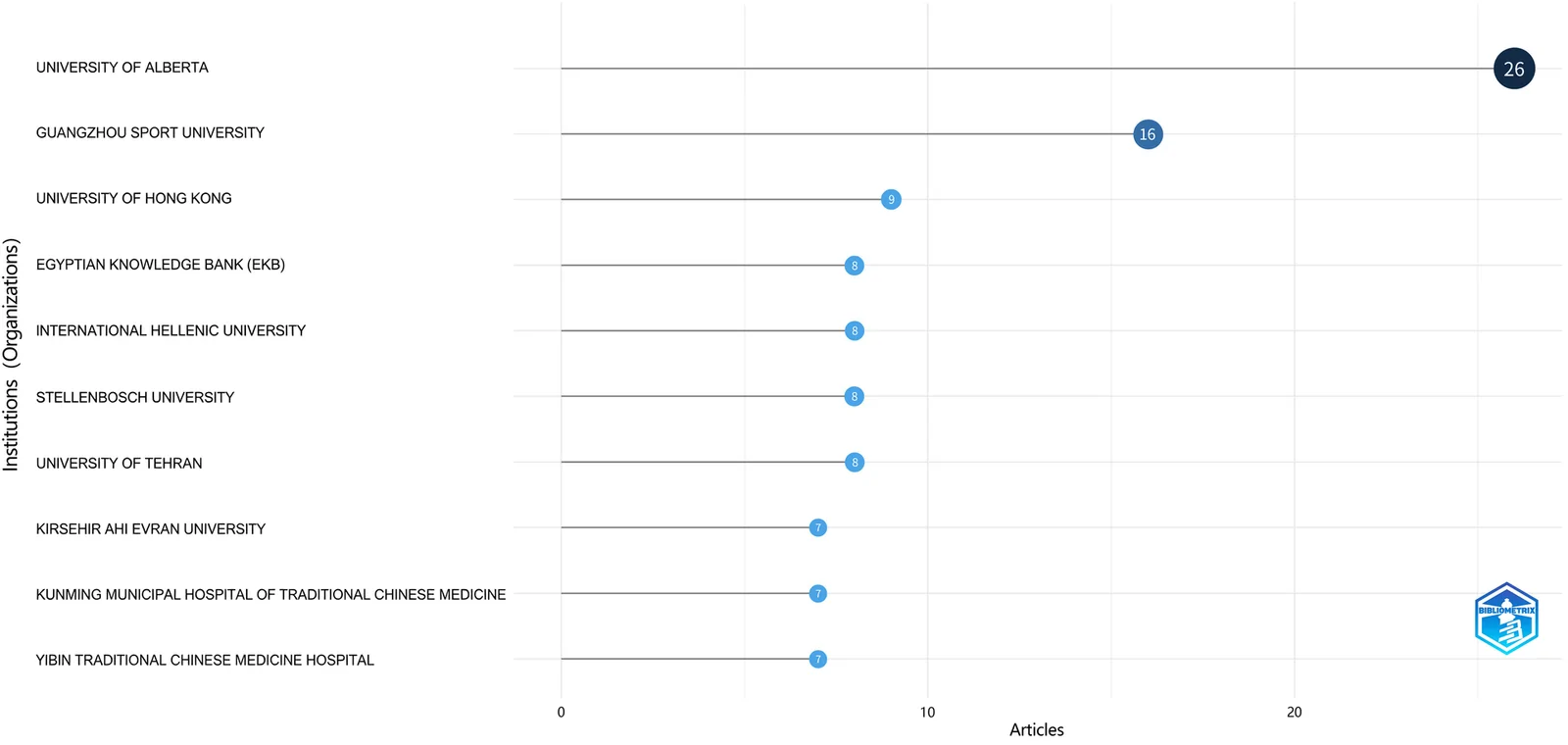
Top 10 institutions by publication output in research on Schroth exercise therapy for AIS. (Egyptian Knowledge Bank was retained as an institution-related metadata entry but was not interpreted as a conventional independent research institution.).

Figure 6 presents the top 10 journals ranked by publication output. BMC Musculoskeletal Disorders ranked first with 8 publications, followed by the Journal of Back and Musculoskeletal Rehabilitation and PLOS ONE, with 6 publications each. Together, these three journals published 20 articles, accounting for 42.6% of the 47 articles represented by the ten journals shown in Figure 6 and 11.6% of the full dataset of 172 publications. Although these were the most productive publication venues, the relatively small proportion of the full dataset indicates that the literature was distributed across a broad range of journals.

**Figure 6:**
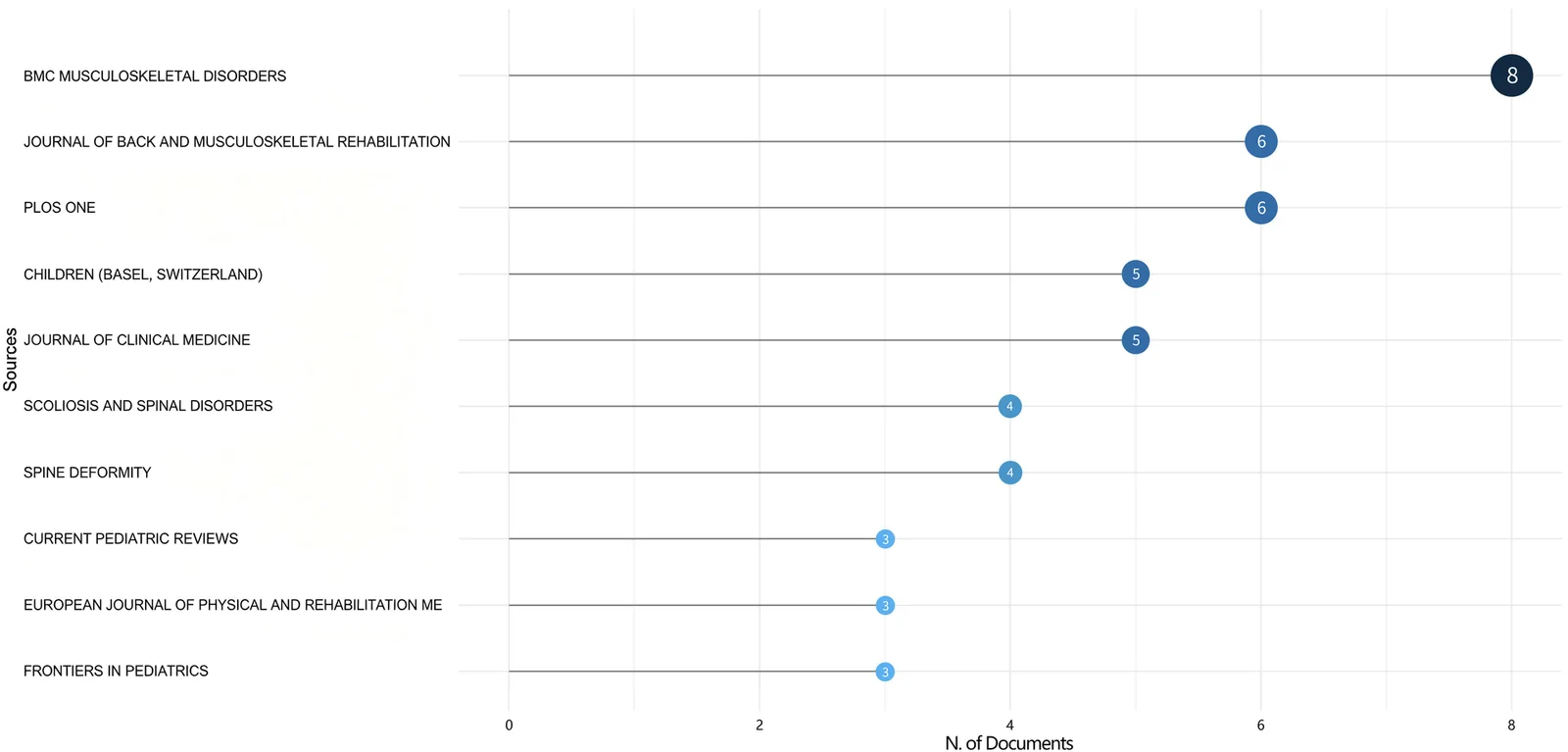
Top 10 journals by publication output in research on Schroth exercise therapy for AIS.

### 3.3 Dual-map overlay of journals and citation pathways

Figure 7 presents a dual-map overlay of journals. The left side represents the disciplinary distribution of citing journals, the right side represents the disciplinary distribution of cited journals, and the curves indicate citation pathways between the two sides. Major citing domains included “Medicine, Medical, Clinical,” “Neurology, Sports, Ophthalmology,” and “Psychology, Education, Health,” whereas prominent cited domains included “Health, Nursing, Medicine,” “Sports, Rehabilitation, Sport,” and “Psychology, Education, Social.”

**Figure 7:**
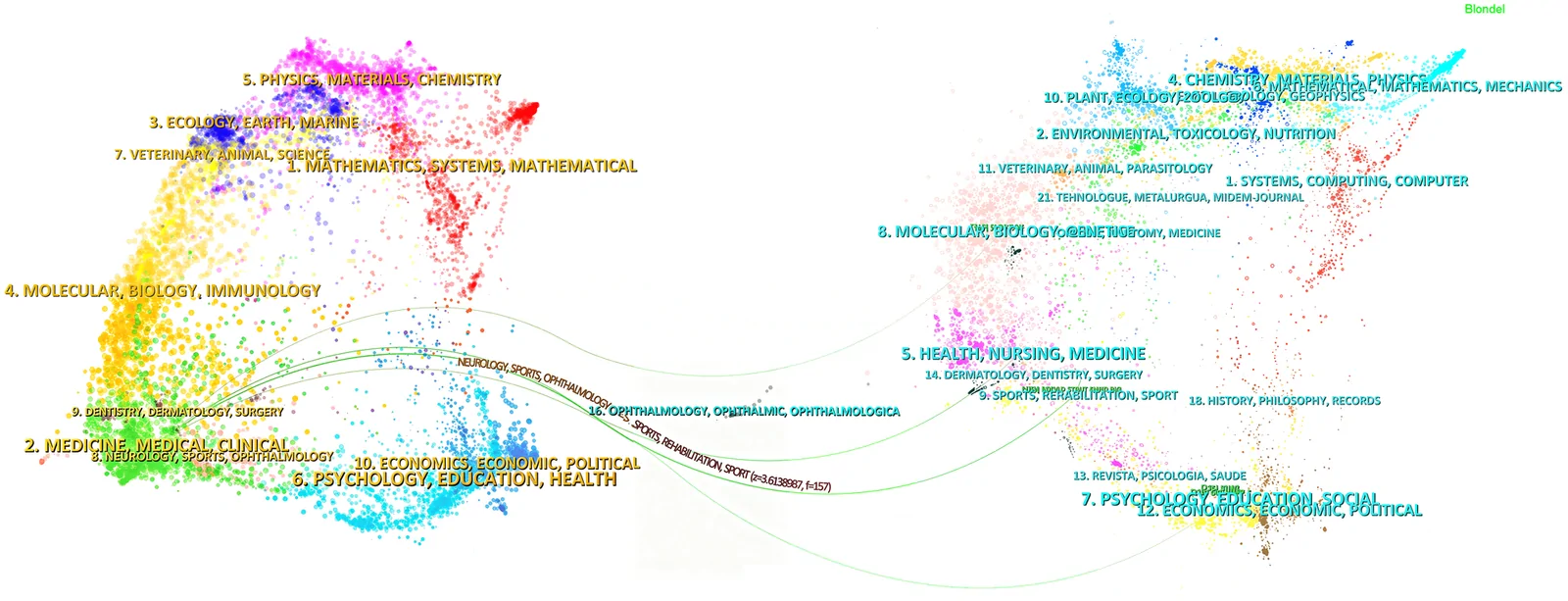
Dual-map overlay of journals and disciplinary citation pathways.

The dominant citation pathways linked clinical medicine and neurology/sports journals to health, nursing, medicine, and sports/rehabilitation journals. These pathways indicate that research on Schroth exercise therapy for AIS is primarily grounded in clinical medicine and rehabilitation knowledge. Additional links involving psychology and education suggest interdisciplinary connections with patient experience, behavior, adherence, and health education (28–30).

Overall, the dual-map overlay demonstrates that the field draws on a clinically oriented and rehabilitation-centered knowledge base while maintaining links with psychosocial and educational research.

### 3.4 Keyword analysis

#### 3.4.1 Clustering, frequency, and centrality of keywords

As shown in Figure 8, the keyword clustering map presents the major research themes related to AIS and their interrelationships. Node colors distinguish clusters, links represent keyword co-occurrence relationships, and node size corresponds to keyword occurrence frequency. The network contained 209 keyword nodes and 316 links, with a density of 0.0145. Cluster validity was supported by modularity Q = 0.6193 and a weighted mean silhouette score S = 0.9055, indicating well-separated and internally coherent clusters.

**Figure 8:**
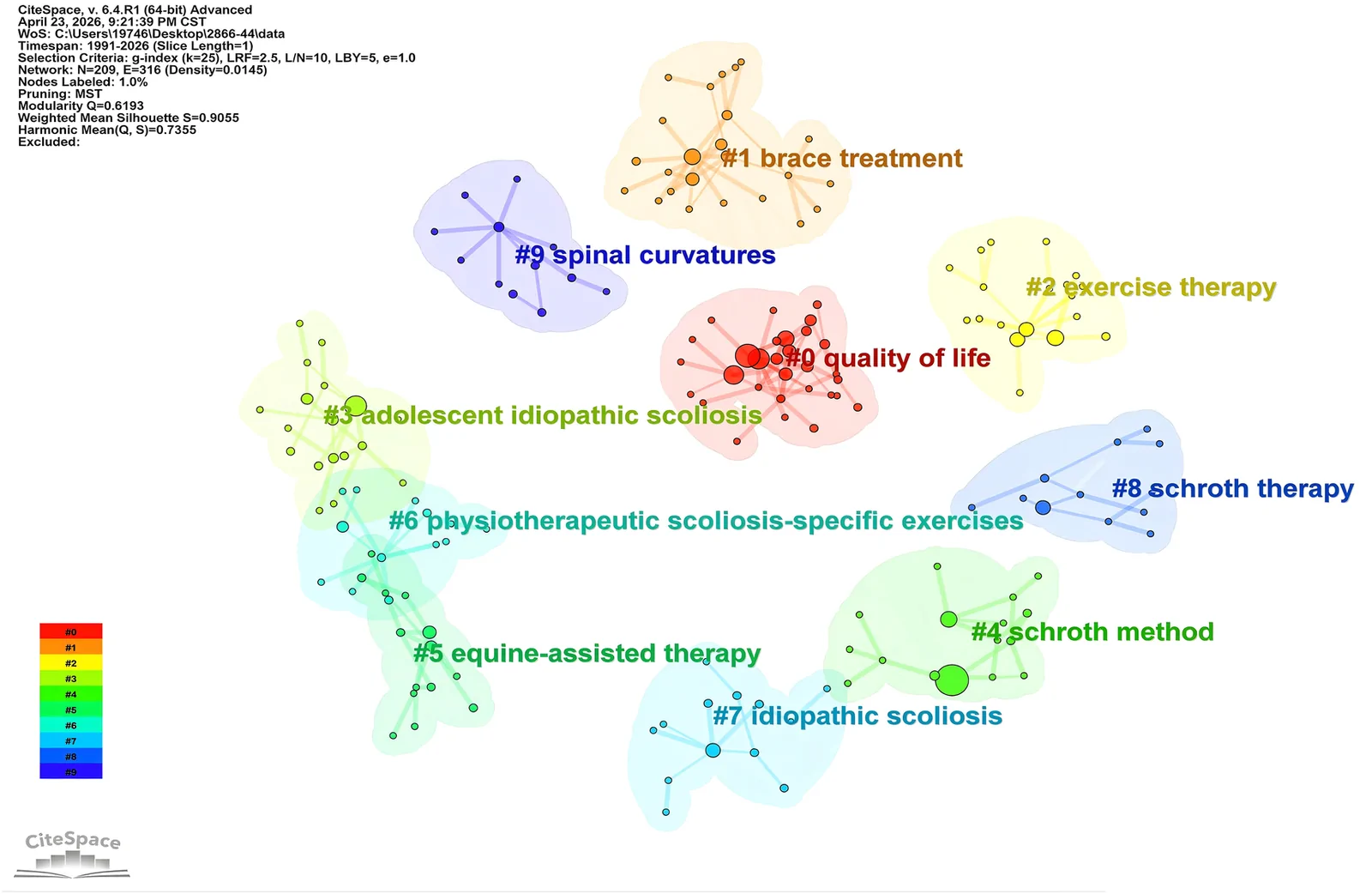
Keyword clustering map.

The analysis identified ten thematic clusters. Cluster #0 was labeled “quality of life,” indicating the importance of patient-reported outcomes and functional well-being. Clusters #1 and #2 were labeled “brace treatment” and “exercise therapy,” respectively, reflecting major conservative intervention strategies. Cluster #3, “adolescent idiopathic scoliosis,” represented the target population and disease category. Cluster #4, “Schroth method,” together with cluster #6, “physiotherapeutic scoliosis-specific exercises,” and cluster #8, “Schroth therapy,” demonstrated a concentrated focus on scoliosis-specific rehabilitation. The remaining clusters—#5 “equine-assisted therapy,” #7 “idiopathic scoliosis,” and #9 “spinal curvatures”—reflected alternative rehabilitation approaches, disease terminology, and structural assessment.

Supplementary Table 1 presents the high-frequency keywords by frequency and centrality. According to Donohue's formula:

$$T=\frac{\sqrt{1+8I}-1}{2}\approx 10.83$$

where I = 64, the occurrence frequency of the most frequent keyword, “adolescent idiopathic scoliosis.” The calculated threshold was approximately 10.83; therefore, keywords occurring at least 11 times were defined as high-frequency keywords. Thirteen keywords met this threshold and were included in Supplementary Table 1.

Among the high-frequency keywords, “adolescent idiopathic scoliosis” had the highest occurrence frequency (64), followed by “Cobb angle” (35), “quality of life” (27), and “efficacy” (27). Frequency reflects the prominence of a term within the dataset. The high frequency of “adolescent idiopathic scoliosis” confirms that AIS was the central topic of the included literature, consistent with previous bibliometric research on scoliosis publications (31).

“Cobb angle,” “quality of life,” and “efficacy” also occurred frequently, indicating sustained attention to structural assessment, patient-reported outcomes, and intervention evaluation. Clinical studies and systematic reviews commonly use Cobb angle as a primary structural measure, while health-related quality of life is frequently evaluated with instruments such as the SRS-22, SRS-24, and SF-36 (32, 33).

By contrast, betweenness centrality reflects the bridging role of a keyword within the co-occurrence network. “Conservative treatment” had the highest centrality (0.29), followed by “quality of life” (0.24), “validity” (0.20), and “Cobb angle” (0.19). The high centrality of “conservative treatment” suggests that it connects exercise therapy, brace treatment, outcome assessment, and broader AIS management themes.

#### 3.4.2 Keyword burst analysis

Figure 9 presents the 15 keywords with the strongest citation bursts between 1991 and 2026. Early bursts included “conservative treatment” (2008–2020), “SEAS.02 exercises” (2008–2014), and “physical exercises” (2008–2014), reflecting sustained attention to conservative management and exercise-based interventions during that period, consistent with contemporary conservative-treatment guidance (34).

**Figure 9:**
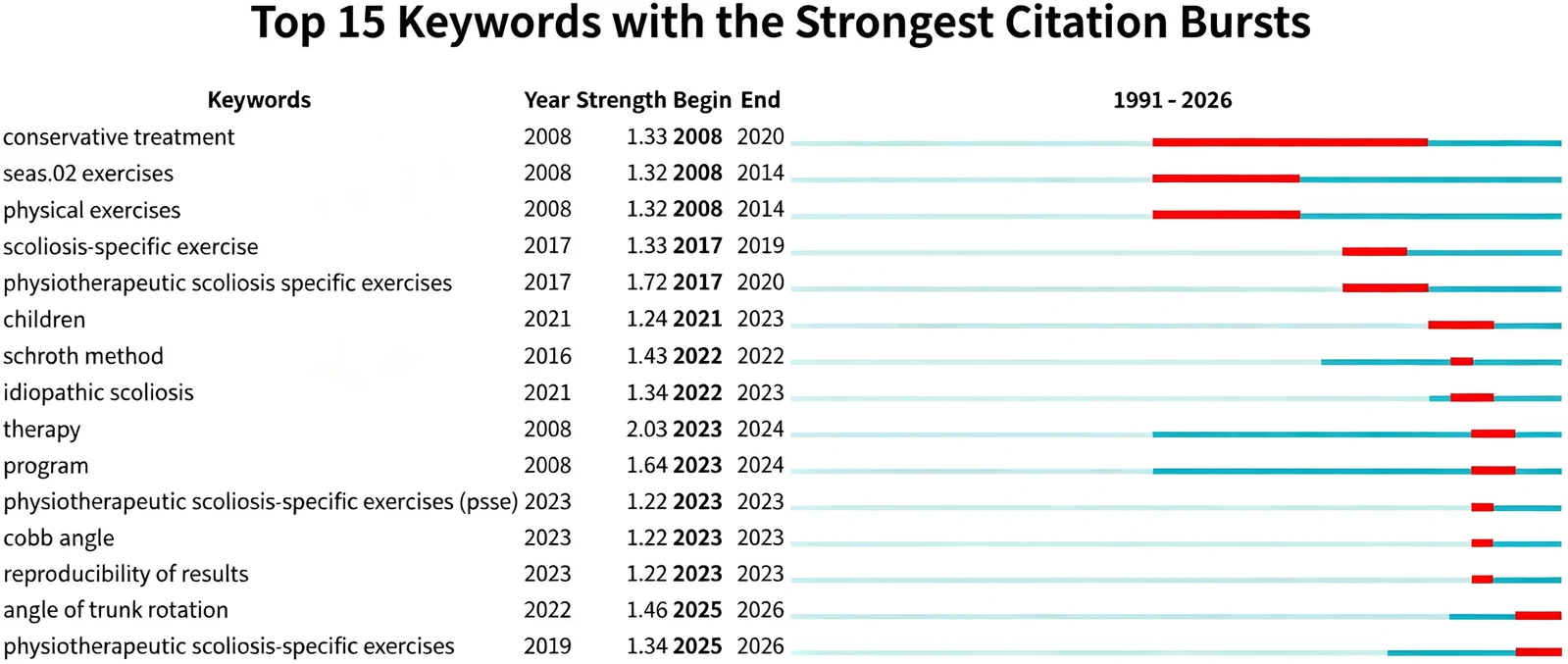
Top 15 keywords with the strongest citation bursts from 1991 to 2026.

After 2017, “scoliosis-specific exercise” (2017–2019) and “physiotherapeutic scoliosis-specific exercises” (2017–2020) showed citation bursts, indicating a shift toward targeted scoliosis-specific rehabilitation. A randomized trial and its subsequent analysis reported that adding Schroth PSSE to standard of care—observation or bracing—improved Cobb angle outcomes and reduced the risk of curve deterioration compared with standard of care alone (20, 35). These clinical studies provide context for the increased bibliometric attention to targeted exercise approaches.

From 2021 to 2024, bursts were observed for “children” (2021–2023), “Schroth method” (2022), “idiopathic scoliosis” (2022–2023), “therapy” (2023–2024), and “program” (2023–2024). These patterns indicate renewed attention to pediatric populations, disease-specific interventions, and structured treatment programs. This period also coincided with accumulating evidence on Schroth-related outcomes, including Cobb angle, ATR, and quality of life (14, 36).

In 2023, short-term bursts were observed for “physiotherapeutic scoliosis-specific exercises (PSSE),” “Cobb angle,” and “reproducibility of results,” indicating increasing attention to quantitative outcome assessment and reproducibility. The use of Cobb angle and ATR together with functional and quality-of-life measures supports comparison of intervention outcomes across studies (14).

The most recent bursts were “angle of trunk rotation” and “physiotherapeutic scoliosis-specific exercises” in 2025–2026, suggesting continued attention to precise outcome assessment and targeted scoliosis-specific interventions. Because the search ended on April 1, 2026, the 2026 burst period represents only a partial year and should be interpreted cautiously.

#### 3.4.3 Keyword timeline analysis

Figure 10 illustrates the temporal evolution of major keyword clusters from 1991 to 2026. Each horizontal line represents a thematic cluster. Node color indicates the time of occurrence, node size represents keyword occurrence frequency, and connecting arcs indicate co-occurrence relationships over time. The network contained 209 nodes and 316 links, with a density of 0.0145. Although the overall network was relatively sparse, coherent thematic clusters and temporal connections were evident.

**Figure 10:**
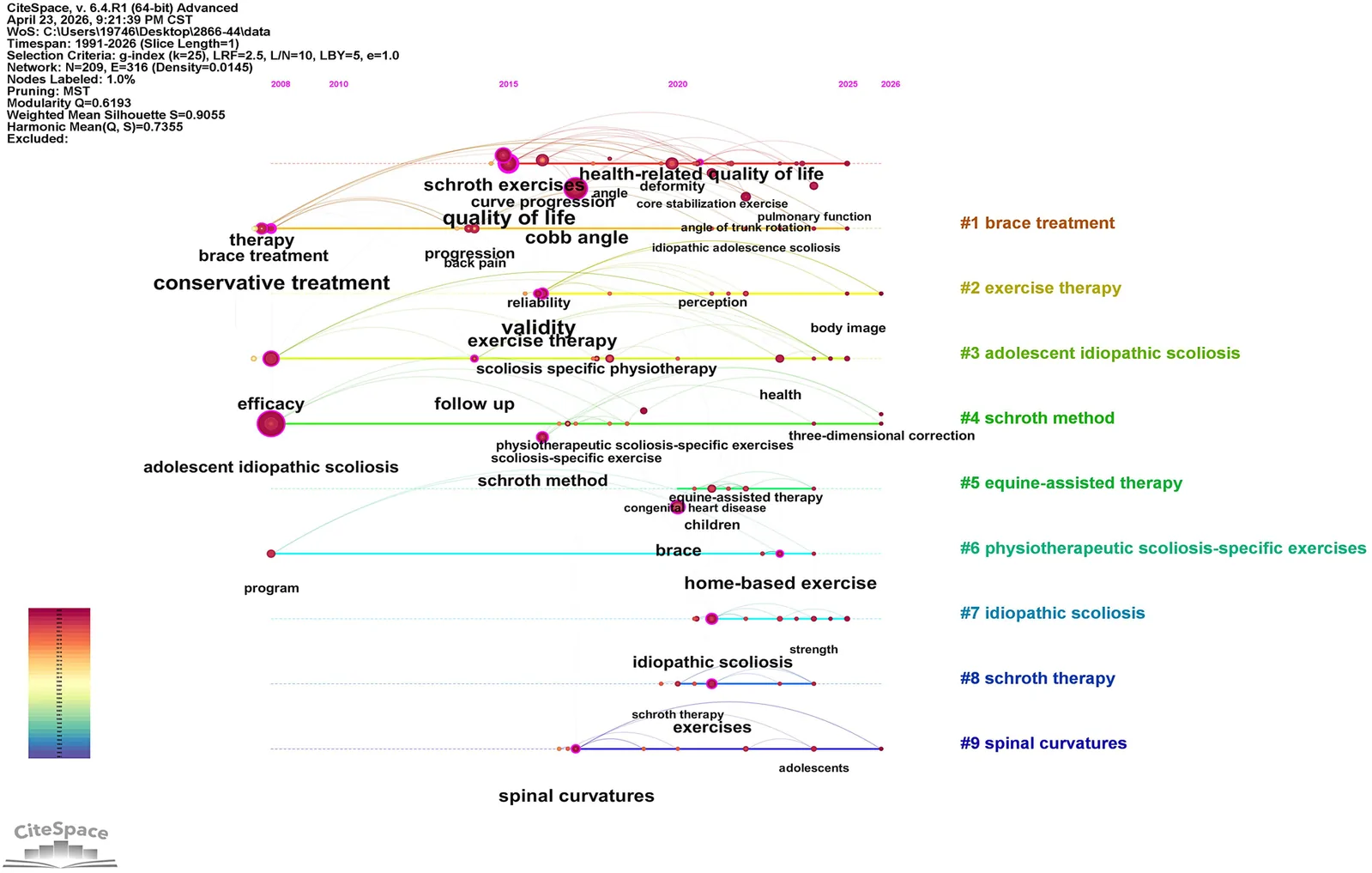
Keyword timeline map showing the temporal evolution of major keyword clusters.

The clusters exhibited distinct temporal patterns. Before 2008, “conservative treatment” and “brace treatment” were prominent. Subsequently, “exercise therapy” and “scoliosis-specific exercises” became more active, reflecting a shift toward targeted rehabilitation. In recent years, terms including “Schroth method,” “Cobb angle,” “angle of trunk rotation,” “health-related quality of life,” and “home-based exercise” have remained active, indicating growing attention to intervention-specific evaluation, quantitative outcomes, and alternative delivery models.

Overall, the timeline analysis shows an evolution from broad conservative-management themes toward Schroth-specific interventions, multidimensional outcome assessment, and home-based rehabilitation.

### 3.5 Author co-citation analysis

Author co-citation analysis was conducted using cited-author records to identify intellectual influence and the knowledge structure of the field. As shown in Figure 11, the network contained 330 cited authors and 1,487 links, with a density of 0.0274. Node size represents co-citation frequency, and links represent co-citation relationships among cited authors.

**Figure 11:**
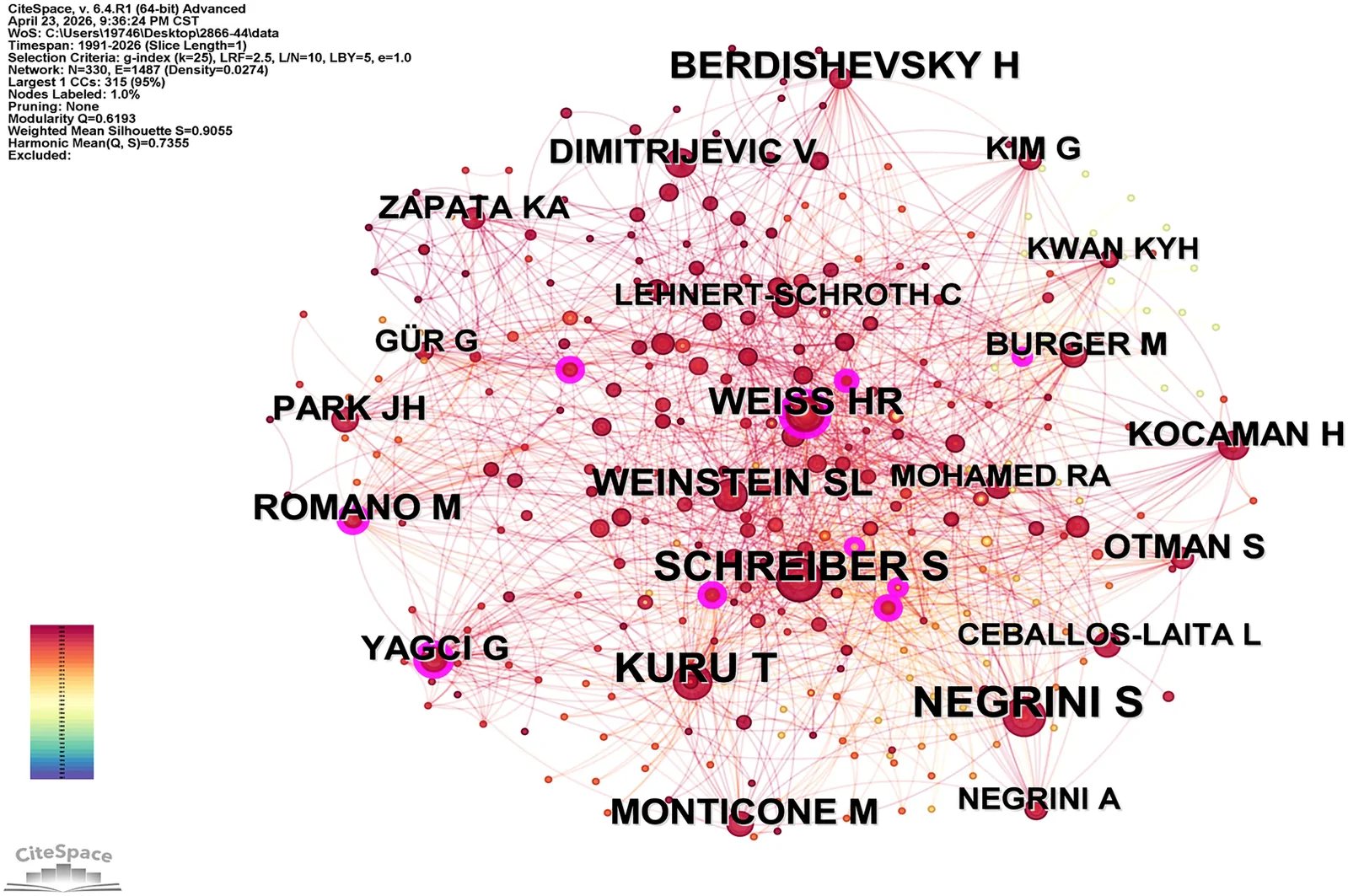
Author co-citation network. (Node size represents co-citation frequency, and links represent co-citation relationships among cited authors.).

Weiss HR, Schreiber S, Weinstein SL, Kuru T, and Negrini S were located near the center of the network or had high co-citation frequencies, indicating substantial intellectual influence in AIS conservative treatment and Schroth-related research. Peripheral nodes and smaller clusters represented cited authors associated with more specific subtopics. Overall, the network showed a connected but differentiated intellectual structure.

Supplementary Table 2 presents the leading co-cited authors. Negrini S, Kuru T, and Schreiber S ranked highest by co-citation frequency, with 62, 58, and 57 co-citations, respectively. Betweenness centrality indicates the extent to which a cited author bridges different intellectual clusters. Yagci G, Weiss HR, and Romano M had relatively high centrality values, suggesting bridging roles within the cited-author network.

### 3.6 Author co-authorship analysis

A co-authorship network was constructed to identify active publishing authors and direct collaboration patterns. As shown in Figure 12, the network contained 297 authors and 498 links, with a density of 0.0113. Node size represents publication output, and links represent direct co-authorship relationships among publishing authors.

**Figure 12:**
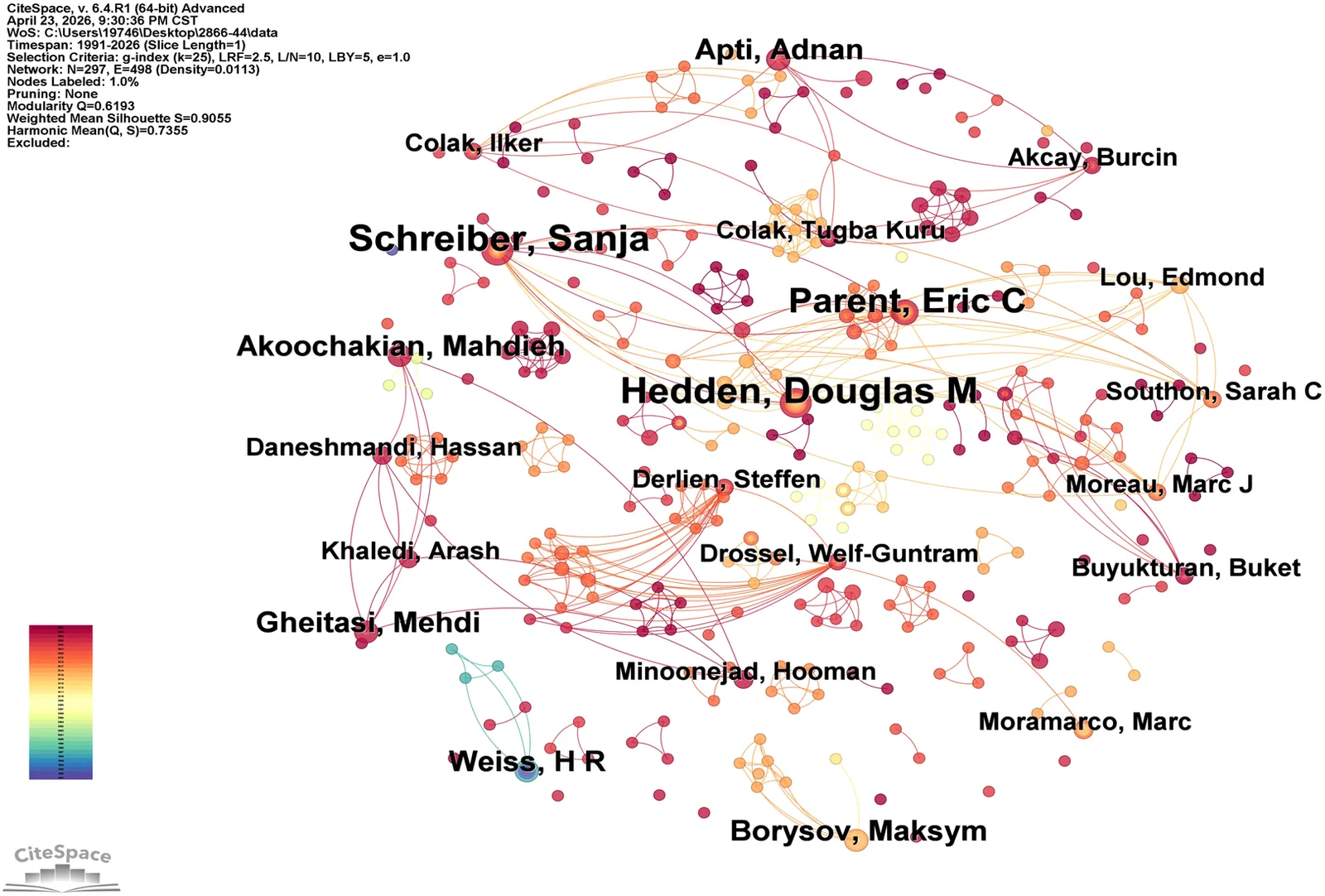
Author co-authorship network. (Node size represents publication output, and links represent direct co-authorship relationships among publishing authors.).

Hedden, Douglas M., Schreiber, Sanja, and Parent, Eric C. had relatively high publication output, while Hedden, Douglas M., Colak, Ilker, and Apti, Adnan had relatively high betweenness centrality. These findings distinguish publication productivity from bridging roles within collaborative groups. Peripheral nodes and smaller clusters indicate several topic-specific or team-based collaboration groups.

Supplementary Table 3 presents the leading publishing authors in the author co-authorship network. Hedden, Douglas M. and Schreiber, Sanja each published 7 articles, followed by Parent, Eric C. with 6. The centrality values indicate the extent to which these authors occupy bridging positions in the collaboration network.

For the co-authorship output analysis, Price’s Law was used to identify core publishing authors:

$$m=0.749\times \sqrt{n_{\max}}$$

where nmax is the publication output of the most prolific author and m is the minimum threshold for identifying core authors. Hedden, Douglas M. and Schreiber, Sanja each published 7 articles; therefore, n_max_ = 7 and m = 0.749 × √7 ≈ 1.98. To make the operational cut-off reproducible, m was rounded to the nearest integer (2), after which a strict greater-than rule was applied. Therefore, authors with publication counts greater than 2 were classified as core publishing authors. Under this rule, 21 authors were classified as core authors, and their individual publication counts summed to 79 author-publication occurrences.

## 4 Discussion

### 4.1 Current status of Schroth exercise research

Bibliometric maps indicate that China had the highest publication output and betweenness centrality, while the United States and Germany were also major contributors by publication output. The United States showed active collaborative links, particularly with China and several European countries; however, its centrality was lower than that of Germany and Canada. These findings demonstrate that publication output, collaboration intensity, and network centrality represent distinct dimensions of country-level contribution.

At the institutional level, the University of Alberta, Guangzhou Sport University, and The University of Hong Kong were the leading conventional research institutions. Author co-citation analysis identified Negrini S, Kuru T, and Schreiber S as influential co-cited authors, whereas co-authorship analysis identified Hedden, Douglas M., Schreiber, Sanja, and Parent, Eric C. as leading publishing authors. Highly cited documents were mainly concerned with intervention evaluation and clinical follow-up. Keyword burst and timeline analyses showed an evolution from broad themes such as “conservative treatment” and “physical exercises” toward Schroth-specific approaches and greater attention to outcomes such as Cobb angle, ATR, and quality of life. This evolution reflects a broader shift from general conservative management toward intervention-specific and outcome-oriented research.

### 4.2 Research hotspots and clinical context of Schroth exercise

Keyword clustering and highly cited clinical literature indicate that current research on Schroth exercise in AIS focuses on intervention effects, multidimensional outcome evaluation, and diversified delivery models. Major themes include three-dimensional correction, functional rehabilitation, Cobb angle, ATR, quality of life, combined treatment, and home-based rehabilitation.

An earlier systematic review and meta-analysis contributed to research interest in Schroth-related outcomes (37). Dong et al. reported that, compared with conventional rehabilitation therapy, PSSE was associated with improvements in Cobb angle [standardized mean difference (SMD) = −0.70, 95% confidence interval (CI) −0.95 to −0.44], ATR (SMD = −1.05, 95% CI −1.52 to −0.58), and quality of life (SMD = 0.61, 95% CI 0.16 to 1.07) (38). Chen et al. reported that Schroth three-dimensional exercise reduced Cobb angle [weighted mean difference (WMD) = −3.32, 95% CI −4.15 to −2.50] and ATR (WMD = −2.24, 95% CI −3.00 to −1.48), while improving quality of life (SMD = 2.80, 95% CI 1.53 to 4.06), Walter Reed Visual Assessment Scale scores (WMD = −2.92, 95% CI −3.25 to −2.60), and lumbar extensor strength (SMD = 1.79, 95% CI 1.46 to 2.12) compared with conventional physical therapy (14). A network meta-analysis of 16 RCTs further reported improvements in Cobb angle (SMD = −1.42, 95% CI −2.03 to −0.80) and ATR (SMD = −1.86, 95% CI −3.05 to −0.68) with Schroth exercise, whereas the Lyon method ranked highest for quality-of-life improvement (SMD = 2.64, 95% CI 0.80 to 4.49) (39).

At the individual-study level, a randomized trial and its subsequent analysis reported that adding Schroth PSSE to standard of care improved Cobb angle outcomes and reduced the risk of curve deterioration compared with standard of care alone, which consisted of observation or bracing (20, 35). A systematic review suggested potential benefit from combining Schroth exercise with brace treatment in mild-to-moderate AIS (40), and a 2024 RCT reported improvements in Cobb angle, ATR, and quality of life after 12 weeks of Schroth exercise combined with asymmetric spinal stabilization training (41). Other comparative evidence has evaluated different scoliosis-specific exercise approaches (42), compared Schroth exercise with proprioceptive neuromuscular facilitation in a recent RCT (43), and highlighted limitations in evidence quality and long-term follow-up (44). Remote rehabilitation is also emerging: Wang and Su examined a six-month, fully online, home-based Schroth–Pilates program delivered alongside brace treatment, providing clinical context for tele-rehabilitation and alternative delivery models (45).

### 4.3 Future trends and limitations of Schroth exercise research

Future research will likely emphasize evidence quality, individualized intervention, multidisciplinary care, and standardized assessment. Timeline analysis showed that early work focused on “conservative treatment” and “brace treatment”; later activity centered on “exercise therapy” and “scoliosis-specific exercises”; and more recent terms included “Schroth method,” “Cobb angle,” “angle of trunk rotation,” “health-related quality of life,” and “home-based exercise.” These patterns indicate increasing attention to intervention-specific evaluation, personalized rehabilitation, and alternative delivery models. Because the search ended on April 1, 2026, patterns attributed to 2026 represent only a partial publication year.

Systematic evaluations of current evidence also indicate priorities for future research. Zhu et al. reported that small sample sizes, heterogeneity, and risk of bias require cautious interpretation and larger, rigorously designed RCTs (39). Intervention models are becoming increasingly diversified: home-based online exercise has emerged as an area of investigation (45), a recent protocol proposed traditional Chinese medicine manipulation combined with Schroth exercise (46), and manual therapy has also been explored as an adjunctive conservative approach (47). Digital delivery and remote supervision may support individualized home-based rehabilitation, but controlled and longitudinal studies are needed. International collaboration patterns further suggest opportunities for cross-regional and multicenter research. Overall, the field is moving from isolated efficacy assessments toward standardized, evidence-supported programs and multidisciplinary rehabilitation models.

Several limitations should be considered. First, the clinical evidence underlying the bibliometric hotspots includes relatively small samples, heterogeneous intervention protocols, and limited long-term follow-up, making sustained effects and subgroup differences difficult to establish (9, 44). Second, outcome measures and instrument versions vary across studies, limiting comparability. Third, only English-language publications indexed in WoSCC, PubMed, and Scopus were included; relevant non-English or regionally indexed literature may therefore have been missed.

Fourth, global citation counts may favor older publications because they have had more time to accumulate citations. Fifth, differences in database coverage and metadata structure may have introduced inconsistencies during data integration. Although author and institution names were checked and standardized where possible, residual name variants may have affected network construction. Finally, 2026 was represented only through April 1, so the most recent publication and burst patterns should be interpreted cautiously.

Future research should (1): conduct large, multicenter RCTs with standardized intervention protocols, long-term follow-up, and harmonized primary outcomes such as Cobb angle, ATR, and quality of life; (2) clarify dose–response relationships and differential effects across curve types and skeletal-maturity subgroups; and (3) investigate mechanisms involving three-dimensional correction, breathing patterns, and neuromuscular re-education to support more precise individualized rehabilitation.

## 5 Conclusion

This bibliometric analysis characterizes the development and research trends of Schroth exercise therapy for AIS. The literature has evolved from broad conservative-treatment themes toward intervention-specific approaches, multidimensional outcome evaluation, and home-based delivery models. The study maps publication output, collaboration patterns, influential documents, leading institutions and journals, influential co-cited authors, active publishing authors, and emerging topics; it does not directly test clinical efficacy. Future work should prioritize rigorous multicenter trials, standardized outcomes, long-term follow-up, and mechanistic investigation.

## Data Availability

The original contributions presented in the study are included in the article and its Supplementary Material. Further inquiries can be directed to the corresponding authors.
