# Supplementary material for: Application of Schroth exercise therapy in adolescent idiopathic scoliosis: a bibliometric analysis
Source: Front Sports Act Living. 2026 Sep 14;8:1872364. doi: 10.3389/fspor.2026.1872364 (PMC13616696; doi:10.3389/fspor.2026.1872364)
Supplement: Supplementary file 1 [file Datasheet1.pdf]

Table 1. High-frequency keywords by frequency and centrality.

Rank	Year	Keywords	Frequency	Centrality
1	2008	adolescent idiopathic scoliosis	64	0.15
2	2017	cobb angle	35	0.19
3	2015	quality of life	27	0.24
4	2008	efficacy	27	0.10
5	2015	schroth exercises	20	0.16
6	2016	schroth method	15	0.15
7	2016	exercise therapy	14	0.15
8	2008	conservative treatment	14	0.29
9	2020	schroth	13	0.15
10	2021	idiopathic scoliosis	12	0.18
11	2021	exercises	11	0.11
12	2016	reliability	11	0.03
13	2016	validity	11	0.20
