# Supplementary material for: Application of Schroth exercise therapy in adolescent idiopathic scoliosis: a bibliometric analysis
Source: Front Sports Act Living. 2026 Sep 14;8:1872364. doi: 10.3389/fspor.2026.1872364 (PMC13616696; doi:10.3389/fspor.2026.1872364)
Supplement: Supplementary file 2 [file Datasheet2.pdf]

Table 2. Leading cited authors in the author co-citation network.

Rank	Cited author	Co-citation frequency	Centrality
1	Negrini S	62	0.09
2	Kuru T	58	0.02
3	Schreiber S	57	0.02
4	Berdishevsky H	37	0.07
5	Weiss HR	35	0.12
6	Weinstein SL	34	0.03
7	Monticone M	31	0.04
8	Romano M	28	0.12
9	Park JH	26	0.03
10	Otman S	25	0.08
11	Kocaman H	25	0.02
12	Yagci G	23	0.18
13	Dimitrijevic V	23	0.02
14	Zapata KA	21	0.03
15	Burger M	21	0.08
16	Kim G	20	0.02
17	Gür G	18	0.04
18	Kwan KYH	17	0.07
19	Mohamed RA	15	0.02
20	Negrini A	15	0.03
21	Ceballos-Laita L	15	0.01
