# Supplementary material for: Application of Schroth exercise therapy in adolescent idiopathic scoliosis: a bibliometric analysis
Source: Front Sports Act Living. 2026 Sep 14;8:1872364. doi: 10.3389/fspor.2026.1872364 (PMC13616696; doi:10.3389/fspor.2026.1872364)
Supplement: Supplementary file 3 [file Datasheet3.pdf]

Table 3. Leading publishing authors in the author co-authorship network.

Rank	Author	Publications	Centrality
1	Hedden, Douglas M	7	0.30
2	Schreiber, Sanja	7	0.18
3	Parent, Eric C	6	0.03
4	Apti, Adnan	4	0.19
5	Akoochakian, Mahdieh	4	0.04
6	Borysov, Maksym	4	0.10
7	Weiss, H R	4	0.05
8	Gheitasi, Mehdi	4	0.06
9	Moreau, Marc J	3	0.02
10	Colak, Ilker	3	0.20
11	Buyukturan, Buket	3	0.07
12	Southon, Sarah C	3	0.03
13	Moramarco, Marc	3	0.04
14	Kuru, T	3	0.04
15	Akcay, Burcin	3	0.11
16	Derlien, Steffen	3	0.06
17	Daneshmandi, Hassan	3	0.08
18	Khaledi, Arash	3	0.03
19	Lou, Edmond	3	0.04
20	Minoonejad, Hooman	3	0.04
21	Drossel, Welf-Guntram	3	0.08
